# Certificates of Medical Men to Quack Remedies

**Published:** 1846-10

**Authors:** 


18. *Certificates of Medical Men to Quack Remedies.*—Certificates of medical men to unqualified, ignorant, or unprincipled persons respecting preparations which are either universal, secret, or the objects of patents, have already been severely condemned on many occasions in the pages of *The Lancet.* But the subject is so important in itself, and its ramifications are so extensive and injurious, that very much remains to be said and reasoned regarding it. We hope to see the day when every man will from conscientious feelings, refrain from granting such certificates under any circumstances whatever, and when every one who desires well of his profession, will take the most energetic means to remove any suspicion of collusion with quacks and quackery. If this feeling could be actively aroused in the profession, we should no more see quacks adopt the names of medical men, and forge their certificates to the most destructive and abominable nostrums, nor have to witness otherwise respectable men making themselves ridiculous by testifying to the virtues or harmlessness of the most inconsiderable trifles. We smile at the obsolete power possessed by the ARCHBISHOP OF CANTERBURY, of making his footman or valet a doctor of medicine, irrespective of education, or any other claim to the honour; yet we tolerate the still more mischivous system by which professional men themselves make practitioners in medicine by the grant of certificates. But we hold that every certificate granted respecting medicinal talent or virtue to any extra-professional person or thing, goes as directly towards constituting the ignorant medical practitioners, as the most flagrant abuse of the power vested in the archbishop ever did or could. It is no use mincing about terms or titles; the men we produce below,—HOLLOWAY, HUNT, KIDDLE, NEWBERRY, DA SILVA, COCKLE, STIVENS, SMITH, FRANKS, RUSPINI, and the rest, are as much medical practitioners, engaged in obtaining money by prescribing medicines, or medical treatment, as Dr. CHAMBERS or Sir BENJAMIN BRODIE themselves. The more is the scandal that they should, any of them, be able to vaunt medical sanction of any kind to their, in many instances, nefarious proceedings.—*Lancet*, July 25, 1846, in *Medical News.*

